# A higher effort-based paradigm in physical activity and exercise for public health: making the case for a greater emphasis on resistance training

**DOI:** 10.1186/s12889-017-4209-8

**Published:** 2017-04-05

**Authors:** James Steele, James Fisher, Martin Skivington, Chris Dunn, Josh Arnold, Garry Tew, Alan M. Batterham, David Nunan, Jamie M. O’Driscoll, Steven Mann, Chris Beedie, Simon Jobson, Dave Smith, Andrew Vigotsky, Stuart Phillips, Paul Estabrooks, Richard Winett

**Affiliations:** 1 School of Sport, Health, and Social Science, Southampton Solent University, Southampton, SO14 0YN UK; 2 Exercise and Health Sciences Department: Sport, Exercise and Rehabilitation, Northumbria University, Newcastle, NE1 8ST UK; 3 School of Health and Social Care, Teesside University, Middleborough, TS1 3BA UK; 4 Centre for Evidence-Based Medicine, Nuffield Department of Primary |Care Health Sciences, University of Oxford, Oxford, OX2 6GG UK; 5 School of Human and Life Sciences, Canterbury Christ Church University, Kent, CT1 1QU UK; 6 UK Active Research Institute, UK Active, London, WC1R 4HE UK; 7 Department of Sport & Exercise, University of Winchester, Winchester, SO22 4NR UK; 8 Department of Exercise and Sport Science, Manchester Metropolitan University, Crewe, CW1 5DU UK; 9 Department of Biomedical Engineering, Northwestern University, Evanston, IL USA; 10 Department of Kinesiology, McMaster University, Hamilton, ON Canada; 11 College of Public Health, University of Nebraska Medical Centre, Omaha, NE USA; 12 Psychology Department, Virginia Tech, Blacksburg, VA USA

**Keywords:** Physical activity, Exercise, Fitness, Cardiorespiratory, Strength, Muscle, Public health, Morbidity, Mortality

## Abstract

It is well known that physical activity and exercise is associated with a lower risk of a range of morbidities and all-cause mortality. Further, it appears that risk reductions are greater when physical activity and/or exercise is performed at a higher intensity of effort. Why this may be the case is perhaps explained by the accumulating evidence linking physical fitness and performance outcomes (e.g. cardiorespiratory fitness, strength, and muscle mass) also to morbidity and mortality risk. Current guidelines about the performance of moderate/vigorous physical activity using aerobic exercise modes focuses upon the accumulation of a minimum volume of physical activity and/or exercise, and have thus far produced disappointing outcomes. As such there has been increased interest in the use of higher effort physical activity and exercise as being potentially more efficacious. Though there is currently debate as to the effectiveness of public health prescription based around higher effort physical activity and exercise, most discussion around this has focused upon modes considered to be traditionally ‘aerobic’ (e.g. running, cycling, rowing, swimming etc.). A mode customarily performed to a relatively high intensity of effort that we believe has been overlooked is resistance training. Current guidelines do include recommendations to engage in ‘muscle strengthening activities’ though there has been very little emphasis upon these modes in either research or public health effort. As such the purpose of this debate article is to discuss the emerging higher effort paradigm in physical activity and exercise for public health and to make a case for why there should be a greater emphasis placed upon resistance training as a mode in this paradigm shift.

## Background

It is hard to argue against the value of physical activity and/or exercise for health and longevity. Engaging in these behaviors is associated with a reduced risk of all-cause mortality [1, 2], and a dose-response relationship appears to exist between increasing volume (i.e., amount or duration) of physical activity and exercise engaged in and reduced mortality risk [3–5]. As a result most guidelines regarding physical activity and exercise are based upon the accumulation of a minimum volume (i.e. a combination of 30 min of moderate intensity [50–70% of maximum heart rate (MHR)] five times per week AND/OR 20 min of vigorous intensity [70–80% MHR] three times per week).

However, the efficacy of these recommendations could be considered disappointing in view of recent studies showing that only a marginal reduction in morbidity risk factors and all-cause mortality occurs when they are met [6, 7]. In contrast, the intensity of effort (i.e. relative challenge) of physical activity and exercise may be a more impactful moderator of risk reduction than exercise volume [8, 9]. Although a combined approach (i.e., higher volumes of low effort exercise combined with lower volumes of high effort exercise) may offer the most benefit, in isolation, engaging in higher effort physical activity and exercise would appear most impactful [10]. It is important to note that most evidence for the benefits of physical activity and exercise comes from observational studies and that evidence is mixed amongst randomised controlled trials and systematic reviews [11]. Despite this uncertainty, it is worth considering why the observational evidence seems to support engagement in higher effort exercise as being more efficacious compared with lower effort yet higher volume approaches.

Evidence is accumulating that poor performance in fitness related measures, across the lifespan, may be some of the strongest risk factors for quality of life, function, and increased risk of a range of morbidities, as well as increased all-cause mortality. The now classic work of Blair et al. [12] reported that cardiorespiratory fitness is a stronger predictor of mortality than even smoking. More recent studies support similar relationships between health, longevity, and cardiorespiratory fitness [6, 7, 13–19], in addition to other characteristics notably modifiable through physical activity and exercise such as muscle mass [20, 21], and strength [22–32]. Considering that these variables (cardiorespiratory fitness, strength and muscle mass) are strong predictors of morbidity and mortality, from the perspective of an exercise physiologist, it might appear unsurprising that higher effort physical activity and exercise also appears to be a strong predictor compared with higher volume, lower effort physical activity and exercise. The use of exercise interventions with high intensity of effort has shown promising efficacy in improving outcomes for a range of cardiometabolic diseases, and may also be superior to moderate intensity of effort programmes at improving outcomes such as cardiorespiratory fitness [33–35]. Indeed, in an experimental examination of the current aerobic physical activity guidelines, Church et al. [36] had groups of participants perform varying volumes of exercise. Participants exercised at an average of ~3.6METs, considered ‘moderate’ activity. Even in the group exceeding the volume of the current guidelines by 50% there was minimal to no effect on a range of risk factors for coronary heart disease, including cardiorespiratory fitness [36].

A paradigm shift is beginning with many now discussing exercise prescription for public health based on an effort driven model (i.e., the prescription of exercise at higher or near maximal relative efforts), and thus a wider range of exercise options to increase reach to a broader and more representative portion of the population. This is evident by the fact that the concept is being taken seriously enough to be the subject of debate at international conferences [37], in addition to the increasing number of studies being funded and published examining the applications of higher intensity of effort interventions for an increasing range of conditions. However, most of the focus around this area has been primarily upon what are often colloquially termed ‘cardio’ exercise modalities (i.e. locomotive based modes such as cycling, running, rowing, incline walking, and stairclimbing). Indeed, though an effort driven model opens up options for exercise, a mode which the authors of this paper believe has been underappreciated and received less discussion in the wider field of physical activity and exercise for public health is resistance training (RT).

## Resistance training for public health

RT is a modality of exercise that has existed in many forms. As early as 480 BC Greek soldiers engaged in a form of RT, often referred to as calisthenics, using their bodyweight to provide resistance during exercise. The use of calisthenics based RT reached a peak in the early nineteenth century with the various gymnastic schools, most notably the Swedish school of Per Henrick Ling. The notion of applying progression to RT by using increasingly heavier forms of external resistance finds its origins in the myth of Milo of Croton who was said to have carried a bull across his shoulders after having lifted it as a new-born calf every day until its maturity. Free weights, such as barbells and dumbbells, are a type of external resistance with which most are familiar today, and the modern adjustable incarnations of these implements came into popularity through the Milo Barbell company, founded by Alan Calvert in 1902. Machines to provide adjustable external resistance are now also commonplace in most gyms and fitness centres. The first designs for such devices are credited to Gustav Zander in the late nineteenth century, though their resurgence and current popularity find their source in the Nautilus Sport/Medical Industries Company founded by Arthur Jones in the 1970s. Many varied forms of RT exist nowadays, the list above not being exhaustive, yet there are some key defining characteristics of how RT is commonly recommended and applied that characterise and differentiate it from other exercise modes. These include repeated or sustained muscular actions against some form of resistance, at a relatively high effort, for a relatively brief duration, and relatively infrequently. Notably RT improves both strength and muscle mass with effort being a primary determinant of these outcomes [38, 39]. Moreover, RT may also improve cardiorespiratory fitness, particularly if performed to a high enough intensity of effort [40].

Evidence has accumulated that suggests that engaging in some form of muscle strengthening activity, such as RT, has an impact on a range of health and morbidity related risk factors [41–45], multi-morbidity risk [46, 47], and all-cause mortality [48–50], across both healthy and clinical populations. However, a question remains as to how important a place RT should have in current physical activity and exercise guidelines for public health. Within the academic literature numerous authors have argued that RT should have a more prominent place within guidelines [51–53]. In fact, most current activity guidelines around the world already include recommendations to engage in some form of muscle strengthening activity at least twice per week [54–58]. Despite this, as Strain et al. [59] noted recently, these are more often than not the ‘forgotten’ portion of the guidelines. However, in addition to the lack of focus in public health policy, we have further concerns with the current state of these recommendations, particularly from the perspective of RT as a higher effort mode of exercise. Recommendations for what constitutes a muscle strengthening activity, considering the potential importance of high effort in moderating efficacy, could be considered as insufficient except in the most unfit of persons. For example, the UK National Health Service recommends the following: lifting weights, working with resistance bands, doing exercises that use your own bodyweight, such as push-ups and sit-ups, heavy gardening such as digging and shovelling, and yoga. The first three of these examples would likely be considered to meet our conceptualisation of RT as a relatively high effort activity. Nevertheless, the inclusion of low resistance, and thus possibly lower effort activities, such as gardening and yoga, could be considered questionable. Though Ekblom-Bak et al. [60] have reported that non-exercise physical activities (NEPA) such as gardening, home/car maintenance, and housework may contribute to improved health and longevity independent of other directed exercise, their examination of NEPA was based on frequency of participation and included a range of activities that might vary in both volume and intensity of effort. Others have reported that many, and in particular women, consider domestic activities to contribute to their moderate to vigorous physical activity, yet such activities are negatively associated with body composition, suggesting they may be insufficient in providing the benefits normally associated with physical activity and exercise [61]. Considering yoga, though participation may be efficacious in older adults [62, 63], possibly due to it requiring a greater relative intensity of effort in this population, a recent study found that after adjusting for age, yoga participation was not associated with a reduced all-cause mortality risk [64]. Again, this might be attributed to yoga presenting an insufficient stimulus with regards to effort in many populations. In fact, studies which have compared groups completing RT based interventions to control groups performing a range of low effort exercises, including yoga, report significant improvements in most health and fitness related outcomes for RT, yet little to no change in controls [65, 66]. Further, these studies were in disabled, older, female cardiac patients where activities such as yoga might be considered to present a relatively greater effort than in most persons.

Merely ‘going through the motions’ by participating in some of the suggested muscle strengthening activities may not produce the desired outcomes. Yet outcomes are what matter to stakeholders, including public health commissioners and policy makers [37, 67]. A recent study comparing the behaviour (i.e. meeting the muscle strengthening activity guidelines), to the outcome of that behaviour (i.e., strength), upon all-cause mortality supports just that. Dankel and colleagues [68] found that those meeting the guidelines but who were not in the top quartile for strength did not have a significant reduction in all-cause mortality risk. Those who were in the top quartile for strength but did not meet the guidelines (i.e., persons that could be considered ‘naturally strong’) had a ~ 46% risk reduction. But, more tellingly, those who met the guidelines and were in the top quartile for strength had a ~ 72% risk reduction. Though observational in nature, this last group could be considered as those most likely to already be engaged in efficacious muscle strengthening activities e.g. RT. Evidently it is imperative that clear instructions regarding the application of appropriate effort during RT activities are implemented into public health guidelines. The most recent Canadian guidelines [56] make a greater attempt at specifically recommending participation in RT (resistance machines, free weights, cable pulleys, bands, etc.) without offering suggestions of activities that may lack efficacy.

Why there is such a lack of emphasis upon RT within current public health guidelines may stem from a number of factors. It appears likely that some element of mischaracterisation of what constitutes RT may be influential, as would appear evident by the currently recommended examples of muscle strengthening activities. As a result, there is seemingly lacklustre support for an approach emphasising RT. Indeed the most recent report informing the current UK guidelines noted that:

*“… any statements on the health benefits of strength training and flexibility should be positioned as secondary and less important than the primary message to adults of undertaking at least 150 min of aerobic activity per week. “*([69], pg, 24).

With policy makers claiming that it has little importance, it is unsurprising that participation in RT receives little emphasis. Indeed, albeit anecdotal, it is our experience that, even at sport and exercise medicine conferences where the value of RT for public health has been discussed, many are not even aware that the current guidelines include recommendations for muscle strengthening activity at all. This lack of emphasis may be a factor responsible for the considerably lower proportion of people engaged in RT compared with those meeting the lower effort aerobic physical activity guidelines. Participation in any form of physical activity or exercise is disappointingly low. Statistics for people meeting the aerobic portion of the guidelines vary from ~15–20% [70–74], though Scotland stands out with particularly high proportions of the population (71% of men and 58% of women) meeting guidelines [75]. Indeed, a recent study shows that 31% of men and 24% of women in Scotland also currently meet the muscle strengthening guidelines [59], with similar rates in England of 34 and 24% for men and women respectively [76]. However, the activities included as counting towards ‘muscle strengthening activity’ in the surveillance methods used vary widely. For example, in the latest Scottish survey, ‘Workout at Gym’ or ‘Exercises’ might be considered as most closely reflecting participation in RT as described above. But what these categories constituted was not specified and the former was used to specify both ‘Weight Training’ and ‘Exercise Bike’ participation. In contrast, surveys specifying ‘Weightlifting’ in England report rates as low as 5% for men and 0.9% for women [70]. Though some data evidently suggests that a similar proportion of people meet the aerobic and muscle strengthening activity guidelines, where differences exist these may be due to different surveillance methods used. Indeed, where surveys have more clearly differentiated between these and more specific RT, participation rates are ~5–6% [70, 73]. This is cause for concern, as many may believe that they are already engaging in behaviours constituting efficacious muscle strengthening activities when, in fact, they likely are not.

It should be acknowledged that the lack of emphasis in public health policy is not the only potential culprit for the lack of engagement with RT [77]. As with any physical activity and exercise, there are common barriers to participation and RT might be considered to present its own unique ones. In addition to the commonly cited barrier of time to exercise participation, many also report barriers associated with the accessibility to specialised equipment and/or facilities, such as travel time and costs [78–81]. Barriers to participation are also likely to be population specific. Indeed, in older community dwelling adults, a population for whom RT may be of particular benefit, who cite similar access barriers to those noted above, many cite ongoing pain and injury as primary barriers to participation in RT [82].

The suggestion is that many assume participation in exercise or physical activity requires the use of specialised equipment and/or facilities, in addition to extensive time commitments. Indeed, as noted, though it can be performed without equipment (i.e., bodyweight), RT is commonly performed using some kind of equipment to provide resistance (i.e., free weights, resistance machines, elastic resistance bands, etc.) and organizational recommendations regarding RT prescription often emphasise these approaches [83]. The recommendations provided by these organizations are also often complex, time-consuming, and require heavy loads for resistance. Complexity in their recommendations includes the use of periodisation in addition to the performance of a high volume of exercises performed in multiple sets resulting in a substantial time commitment. However, many of these recommended RT practices have in fact been heavily questioned. Periodisation is lacking in evidence for its efficacy [84, 85], multi-jont exercises appear to offer similar benefits as single joint-exercises for most muscle groups [86], and assuming effort is sufficiently high single-set protocols offer largely similar benefits to multiple-set protocols [38, 39]. Indeed a number of studies provide examples of where a relatively low to moderate dose of RT has been effective for a range of health outcomes for both young and old populations (e.g. [87–95]). Further, many oragnisations also imply in their recommendations of particular relative loads (i.e., % of 1 repetition maximum [RM]) that a readily modifiable external resistance is in fact necessary, which may not be the case [96, 97], with perhaps the exception of for outcomes such as bone mineral density where, though low loads can still produce benefit, higher loads might optmise these outcomes [98]. As such, many are likely unaware that RT can be performed in a time efficient manner in a variety of settings with minimal/no equipment. For example, in Mexican primary care settings it is common to have exercise space and water bottles of various sizes filled with sand for RT activities—materials that are locally available at little or no cost, but can be used in a facility or home environment.

On top of this, despite recent work looking to understand barriers and motivators to participation in RT [82], a theoretical model to guide interventions to increase initiation and adherence is currently lacking [77]. Thus we are currently in a position whereby we have considerable evidence supporting the efficacy of RT (i.e., that it works when people do it under ideal conditions), but a considerable lack of evidence examining its effectiveness (i.e., whether people will actually do it under ecologically valid conditions). At present this is a conundrum for most of sport and exercise medicine [67], though, with its lack of emphasis in public health research, even more so for RT.

## Conclusions

We acknowledge that for many the primary issue relating to physical activity and public health is first and foremost how we can get people to do any in the first place. In this respect there are contrasting opinions and ongoing debate regarding the application of higher effort models of physical activity and exercise to public health [37]. It might therefore seem almost self-indulgent for researchers to opine on the potential benefits of RT in this respect. However, though at present there may be little evidence supporting the effectiveness of ecologically valid approaches to RT for public health, we are quite convinced that at present we have considerable evidence suggesting it may be an efficacious approach. As such, our motivation for penning the present piece is twofold.

First we hope to increase interest in RT such that more care providers might participate in specific conversations about its engagement and participation. Indeed, it has recently been argued that doctors should be able to prescribe exercise like a drug [99] and an effort based model to inform RT prescription would appear to have considerable merits [52]. Few doctors make recommendations for physical activity participation of any kind and in instances when they do they invariably emphasise aerobic exercise (59% of the time) compared with RT (13% of the time) [100]. Further, when systematic approaches to address exercise promotion in clinical settings are developed, screening and exercise promotion messages often do not address RT leaving physicians and patients without basic tools to cue a conversation and goal setting in the area [101, 102]. The power of such conversations to at least heighten awareness of RT as a complementary or even alternative approach, in physical activity and exercise should not be overlooked. The elderly in particular seem receptive to physician’s recommendations in this regard, with this being almost as commonly cited as a motivator for RT participation as knowledge of its health benefits [82].

Appreciably, the above intent is unlikely to translate to a sudden upsurge in public participation in efficacious RT approaches. Nonetheless, our second motivation is that that this piece may serve to stimulate a wider academic interest in RT from a public health perspective, and to highlight the need for trials examining not only the efficacy of this mode, but also the effectiveness. Rigorous trials examining complex interventions – informed by appropriate theoretical models aimed at behavioural change to overcome barriers, increase initiation, and maintain adherence to RT interventions – are essential, in combination with appropriate health outcomes examined as dependent variables (outcomes), as such variables are important to stakeholders and policy makers. In addition to this is a need to identify interventions that are cost effective and sustainable in their implementation. There has been a call for all exercise trials, including RT, to be examined in real world settings such as community centres [103].

Some models already exist for better integrating efficacious RT into public health interventions, including the Lift for Life® RT program in Australia. Recent work has examined the factors associated with engaging in RT behaviours in addition to the application of theory-based approaches for maintaining RT behaviours [82, 104–106], and evaluations of community based interventions are emerging [107]. Thus far, findings have been promising, as they suggest that there are likely simple, low cost, effective approaches possible to increase RT behaviours. We are optimistic that this piece and further work may help to finally push the present higher effort paradigm shift to more explicitly and prominently include RT in its message for the benefit of public health.

## Abbreviations

MHR: Maximum heart rate
NEPA: Non-exercise physical activities
RM: Repetition maximum
RT: Resistance training
